# Identifying Client Targets for Improved Mobilization and Uptake of Integrated Family Planning and Reproductive Health in Environmental Programs in Kenya

**DOI:** 10.3389/fgwh.2021.559297

**Published:** 2021-02-12

**Authors:** Edmon Obat, Krista Schaefer, Mumma Opiyo, George Otieno, Henrietta Windindi, Derick Omuodo, Supriya D. Mehta

**Affiliations:** ^1^Nyanza Reproductive Health Society, Kisumu, Kenya; ^2^Division of Epidemiology & Biostatistics, University of Illinois at Chicago School of Public Health, Chicago, IL, United States

**Keywords:** program evaluation, population, health, and environmental health, Kenya, family planning, reproductive health services, environmental health, health education

## Abstract

**Background:** We conducted a population health environment program in Lake Victoria Basin (LVB) and assessed incorporation and integration of family planning with environmental conservation.

**Methods:** Routine program data were collected from clients by community-based distributors from four environmental community-based organizations. Multivariable regressions identified factors associated with distribution of: (1) oral contraceptive pills to women, (2) male condoms, and (3) integrated family planning and environmental messaging.

**Results:** April 2015 through May 2016, 10,239 client encounters were completed, with 56% made by men. We distributed contraceptive pills at 28% of client encounters. Multivariable modeling showed this was more likely for women <40 years old (*p* < 0.001) and was less likely for women attending household (30%) and group sessions (46%) compared to individual sessions (*p* < 0.001). Male condoms were distributed at 73% of client encounters; (*p* < 0.01, all) women were half as likely to receive condoms than men, and single and widowed clients were more likely than married clients to receive condoms. Integrated messaging occurred at 89% of client encounters, and was 85% more likely for women, increased with client age, and was less likely for single and widowed persons. Exit interviews with 87 clients (42% male, 58% female) confirmed program data by report of commodities received: 27% contraceptive pills, 75% male condoms, 91% integrated messaging.

**Conclusions:** Partnership with environmental conservation organizations effectively expanded family planning and reproductive health to non-traditional audiences and men among rural communities surrounding LVB-Kenya. Specific client subgroups can be targeted for improved mobilization and uptake of services.

## Introduction

Population, health and environment (PHE) capitalizes on the interconnectedness between people's livelihoods, health and the environment to promote family planning (FP) (1). The feasibility of PHE has been demonstrated broadly, including in the Philippines, Ethiopia, Madagascar, Uganda, and Kenya (2, 3), and has demonstrated capacity to reach men and adolescent boys (1, 3). A review of PHE programs from the Philippines, Ethiopia, and Madagascar shows increased uptake of FP over stand-alone or parallel programs (3).

PHE programs are mainly targeted toward remote and ecologically sensitive landscapes where the population often have limited access to FP. The majority of the population living around the Kenyan Lake Victoria Basin (LVB) rely on the land, agriculture and fishing for their livelihoods and the region is characterized by high poverty rates (4). Due to declining land productivity, soil degradation, desertification, loss of biodiversity, and crop and livestock diseases, food security has been challenged (5, 6). With high total fertility rate, 3.9 per woman nationally and 4.3 per woman for the Nyanza Region which encompasses the Kenyan LVB, there is rapid population growth (4). In the LVB counties of Kenya, 20–25% of children are born <24 months after a previous birth (4). The situation is exacerbated by the high unmet need for FP in Nyanza Region (4).

In the most recent Kenyan Demographic Health Survey, modern contraceptive use increased from 32% in 2003 to 53% in 2014 (4), and new data from the Kenyan Ministry of Health shows that modern contraceptive use was at 61.4% in 2019 with 78.4% satisfied demand (7). The most commonly used modern contraceptives are injectables (48%), implants (18%), oral pills (14%), intrauterine device (5.9%), and sterilization (5.6%) (7). Despite this success, modern contraceptive coverage is unequally distributed, with Nyanza Region being an area with lower coverage, especially amongst women in rural areas and those who are impoverished (4). The effects of contraceptive gap are well known: negative impacts on educational attainment for girls and women with subsequent economic impacts, correlation with unsafe abortion, and increased rates of adverse pregnancy outcomes. Drivers of contraceptive gap are multifactorial, but traditional cultural roles and the influence of men's knowledge and beliefs related to FP are critical, making PHE a desirable approach to promoting FP (1–3).

Through the program called SHAPE-LVB (Sustainable Health and People's Environment in Lake Victoria Basin), Nyanza Reproductive Health Society (NRHS) provided technical assistance and training to one non-governmental organization (NGO) and three community-based organizations (CBOs) to enable them to integrate FP into their programs using a PHE approach. While the utility of PHE approach has been demonstrated, evaluations of large-scale programs are limited. We identified client and service delivery factors associated with integration of FP messaging and commodity distribution into routine environmental, health, livelihood and poverty alleviation programs, to better understand how to improve program delivery.

## Materials and Methods

### Description of SHAPE-LVB Program

NRHS is a NGO based in Nyanza Province, Kenya, that focuses on reproductive health services and research including voluntary medical male circumcision, FP, and HIV prevention and treatment. The partner NGO and CBOs provide services to: (1) fisher folk (poverty alleviation, HIV prevention, welfare of children and parents), (2) natural resources management workers (food security, capacity building, water and economic empowerment), (3) agricultural livelihood workers (rainforest preservation, indigenous plant promotion, and energy-saving education), (4) environmental protection workers (plastic waste management). SHAPE-LVB staff trained 48 cross-sector community based distributors (CBDs) (12 from the NGO and each CBO) in providing community-based FP, provision of FP commodities and other PHE related commodities and giving cross-sector referrals for FP methods. From September 2014 through March 2015, SHAPE-LVB staff developed and tested a curriculum based largely on The BALANCED Project methodology in Tanzania (8) and also drawing on the HoPE-LVB program in Uganda and Kenya (9, 10) to integrate FP and reproductive health (RH) messaging with environmental conservation.

We started the program process with participatory appraisal at project sites and PHE context-specific messages based on key informant interviews. Leaders from the NGO and each CBO were trained, and after sufficient performance on post-test assessments, they then trained their organization's CBDs. The CBD's ability was also assessed by pre- and post-test evaluation of knowledge and skills to employ integrated messaging and distribution of FP commodities, specifically condoms and oral contraceptive pills. SHAPE-LVB staff developed a network of providers for referral for long-term contraceptives and obtained supplies from Ministry of Health (MoH) public health facilities. Each CBD was linked to a single nearby health facility and introduced to the facility's leadership, specifically the FP commodities focal person. All local facility and community leaderships were informed about the project and the implementation model. Entry meetings were conducted with all the departmental heads of health of the counties and sub-counties (the sub-national administrative units in Kenya) where the project was implemented. The CBOs and NGO facilitated community entry through meetings with the community gatekeepers. The CBDs then collected the condoms and oral contraceptive pills and replenished their stock as needed, after submission of monthly reports using the MoH tools for dispensing FP commodities. The FP commodities distributed by the CBDs were free of charge to the clients since they were provided by the MoH. CBDs were given a monthly stipend of 1,500 Kenyan shillings (~$15 US dollars) to reimburse them for travel and time. After this phase of training and capacity building, the SHAPE-LVB program was implemented in April 2015.

CBDs met with clients in their communities to discuss FP and environment, and distribute FP commodities. Integrated messaging and data collection were conducted in the clients' preferred language (English, DhoLuo, Kiswahili). Information from these meetings, or client encounters, was documented to help assess the FP integration program, which is the level of analysis. Many CBDs had multiple encounters with a client. At each encounter between the respondents and the CBDs, the respondents were provided with information on the various Family Planning (FP) methods as well as what the CBDs could offer. Additionally, they were offered contextually relevant messaging on environmental conservation measures. Depending on the respondent's choice, they either received the FP of choice from the CBD at that encounter or at a follow-up visit to the respondent's home; or, were referred to the nearest health facility for the methods the CBDs did not provide.

### Data Collection and Program Measures

Self-reported sociodemographic variables were age, sex, marital status, job, and prior FP use were collected by CBDs using closed-ended, standardized forms. CBDs also recorded the session type (individual, family, group), commodities distributed, and the content of messaging discussed. Integrated messaging was defined as receiving at least one of each message (environmental and FP/RH) at a single client encounter. Examples of environmental topics include safe and clean drinking water, forestation, or sustainable fishing; examples of FP/RH topics include modern contraceptive methods, pregnancy spacing, STI/HIV prevention/treatment, and voluntary medical male circumcision. In the last 2 months of the program, a structured exit survey was made available at all sites to measure counseling topics, services received, and satisfaction with services and CBDs, and was administered by NRHS interns (as part of student practica; i.e., they were independent of the SHAPE-LVB program). Interview completion was voluntary and represents a convenience sample of clients.

### Statistical Analysis

The analysis of SHAPE-LVB program data was determined exempt (did not meet the definition of human subject research) by the University of Illinois at Chicago School of Public Health. We identified factors associated with: (1) distribution of any contraception pills (progestin-only or combined progestin and estrogen) to women; and among men and women, (2) distribution of male condoms, and (3) the dissemination of integrated messages. Univariate analysis described the distribution of client encounter factors and the distribution of contraceptive commodities. Bivariate analyses showed the association between the covariates and each outcome variable. Covariates were entered into multivariable analysis if Pearson Chi-squared test was significant at a *p*-value <0.05. Multivariable regression models identified factors associated with distribution of commodities (binomial) and integrated messaging (multinomial). Because clients could have multiple visits over time, analyses were conducted at the encounter-level and we therefore used cluster-based variance estimation to account for correlations among individuals with multiple visits. Gender was assessed for interaction with a likelihood ratio test, and it was included if the *p*-value was <0.05. We conceptualized a multi-level modeling approach to enable us to assess the effects of covariates at different levels on the outcomes because individuals were nested within CBDs and CBDs were nested within CBOs or NGO. We concluded that no random effects terms were needed for CBD or CBO/NGO based on the Hausman specification test (11) at a significance level of *p*-value > 0.05 and minimal difference between fixed effects and random effects coefficients. Therefore, we present fixed effects associations with 95% confidence intervals (95% CI) generated with cluster-based variance. Log-binomial regression and multinomial regression with exponentiation of the logit coefficients generated adjusted prevalence rate ratios (aPRR). There was <1% missing within variables in this data. All analyses were performed in Stata/SE version 14.0 (Stata Corporation, College Station, TX).

## Results

From April 2015 through May 2016, SHAPE-LVB conducted 10,239 client encounters (56.2% men) completed by 6,583 individuals [3,561 men (54.1%), 3,022 women (45.9%)]. Of clients with multiple visits, the median was 2 (range 2 to 15 visits) and did not differ between men and women (*p* = 0.22). The median age of both men and women was 28 (IQR 24–33; Table 1). Two-thirds of clients (66%) were married and over 70% had used FP methods before the encounter. Client sessions were 63% individual, 15% household, and 22% group. The majority of encounters (62%) were with farmers or environmentalists; 10% were with other target populations (7% with fishers, 3% with sex workers).

**Table 1:** Distribution of encounter-level client factors from April 2015–May 2016, *N* = 10,239.

Variables^*^	**Males encounters *N* = 5,753 (56.2%)**	**Female encounters *N* = 4,486 (43.85%)**
	***n* (%)**	***n* (%)**
Median Age in Years (IQR)	28 (24–33)	28 (24–33)
**Marital status**		
Married	3,641 (63.3)	3,092 (68.9)
Single	2,042 (35.5)	1,094 (24.4)
Widowed	70 (1.2)	300 (6.7)
**Used any family planning**		
Ever	4,206 (73.2)	3,244 (72.4)
Never	1,543 (26.8)	1,234 (27.6)
**Session type**		
Individual	3,536 (61.5)	2,947 (65.7)
Household	748 (13.0)	784 (17.5)
Group	1,468 (25.5)	754 (16.8)
**Environmental population served**		
Environmental protection	1,013 (17.6)	1,460 (32.6)
Natural resource Management	1,944 (33.8)	1,061 (23.7)
Agricultural management	1,831 (31.8)	1,143 (25.5)
Fisher Fork support	965 (16.8)	822 (18.3)
**Target population**		
Farmer/Environmental	3,876 (67.5)	2,462 (55.0)
Fisher person	351 (6.1)	359 (8.0)
Other^†^	1,356 (23.6)	1,267 (28.3)
Sex worker	4 (0.1)	242 (5.4)
Student	159 (2.8)	150 (3.4)
Message^**^		
Integrated	5,093 (89.1)	3,978 (89.1)
Reproductive-Health-Only	351 (6.1)	359 (8.0)
Environmental-Only	275 (4.8)	127 (2.8)
**Number of** ***individuals*** **and multiple visits**		
Individuals with one visit only	2,470 (69.4)	2,262 (74.9)
Individuals with two or more visits	1,091 (30.6)	760 (25.1)
Median (Range) Visits per individual with more than one visit	3 (2–15)	2 (2–13)

* *Some cells do not sum to N due to missing data; missing data ranged from 2 to 13 observations (<0.2%), except as otherwise noted*.

** *missing, n = 56 (0.5%)*.

† *Other primarily included boda boda drivers and touts, informal business persons, casual laborers, house help*.

### Factors Associated With Receipt of Contraceptives

We distributed contraceptive pills at 1,239 (28%) client encounters with women (Table 2). Results of multivariable modeling (Table 3) demonstrate that oral contraception was increasingly more likely to be distributed to women until age 30 and then decreased with increasing age (*p* < 0.001). Women attending in household and group sessions were 30 and 46% less likely, respectively, to receive contraceptives than those attending individual sessions (*p* < 0.001). Women who were single or widowed were also less likely to receive contraception than married women (12 and 20%, respectively; *p* < 0.001).

**Table 2:** Encounter-level distribution of commodities from April 2015–May 2016, *N* = 10,239.

Variable^*^	
**Overall**	***N*** **=** **10,239**
	***n*** **(%)**
Distribution of male condoms at all client encounter	7,439 (72.7)
Median number of condoms Received (IQR)	20 (15–30)
Distribution of male condoms at male client encounters	5,385 (93.6)
Median number of condoms received (IQR)	20 (15–30)
Distribution of male condoms at female client encounters	2,054 (45.8)
Median number of condoms received (IQR)	20 (12–30)
**Distribution of commodities at female client encounters**	***N*** **=** **4,486 (43.8%)** ***n*** **(%)**
**Female condoms**	
Any	294 (6.4)
None	4,192 (93.6)
**Receipt of contraceptive pills**	
Yes	1,239 (27.6)
No	3,247 (72.4)
**Progestin only pills, number of months supply**	
0	4,149 (92.5)
1	226 (5.0)
2	14 (0.3)
3+	97 (2.2)
**Combined pill, number of months supply**	
0	3,583 (79.9)
1	600 (13.4)
2	63 (1.4)
3+	240 (5.4)
Referred to health facility for long term contraception^*^	
Yes	398 (8.9)
No	4,080 (91.1)

* *Sum cells do not sum to N due to missing data; missing data ranged from 2 to 8 (<0.2%)*.

**Table 3:** Results of multivariable log-binomial regression: factors associated with receiving contraceptive pills among women.

	**Received contraceptive pills**		**Chi-squared *P*-value**	**Crude PRR (95% CI)**	Adjusted PRR^*^ (95% CI)
					***N*= 4,479**
	**Yes, *N* = 1,239**	**No, *N* = 3,247**			
	***n* (%)**	***n* (%)**			
**Age in years**					
13–19	42 (18.2)	189 (81.8)	<0.01	1.24 (0.85–1.81)	1.30 (0.82–2.06)
20–24	322 (29.0)	790 (71.0)		1.97 (1.50–2.60)	1.90 (1.37–2.63)
25–29	399 (32.6)	825 (67.4)		2.22 (1.69–2.91)	1.89 (1.38–2.60)
30–34	279 (29.0)	682 (71.0)		1.98 (1.50–2.61)	1.76 (1.28–2.43)
35–39	148 (23.7)	476 (76.3)		1.62 (1.20–2.17)	1.39 (1.00–1.95)
40+	49 (14.7)	285 (85.3)		Ref	Ref
**Marital status**					
Married	946 (30.6)	2,146 (69.4)	<0.01	Ref	Ref
Single	232 (21.2)	862 (78.8)		0.69 (0.61–0.79)	0.88 (0.76–1.03)
Widowed	61 (20.3)	239 (79.7)		0.66 (0.53–0.84)	0.80 (0.61–1.05)
**Session type**					
Individual	927 (31.5)	2,020 (68.5)	<0.01	Ref	Ref
Household	195 (24.9)	589 (75.1)		0.79 (0.69–0.90)	0.70 (0.60–0.81)
Group	116 (15.5)	638 (84.6)		0.49 (0.30–0.33)	0.54 (0.45–0.65)
**Family planning usage**					
Ever	845 (26.1)	2,399 (73.9)	<0.01	Ref	N/A, not in final model
Never	392 (31.8)	842 (68.2)		1.22 (1.10–1.35)	
**Environmental population served**					
Environmental protection	248 (17.0)	1,212 (83.0)	<0.01	Ref	Ref
Natural resource management	261 (24.7)	798 (75.4)		1.45 (1.24–1.69)	1.24 (1.00–1.54)
Agricultural management	593 (51.8)	551 (48.2)		3.05 (2.69–3.46)	2.46 (2.05–2.95)
Fisher fork support	137 (16.5)	686 (83.4)		0.98 (0.81–1.19)	0.94 (0.73–1.20)
**Target populations**					
Farmer/environmental	830 (33.7)	1,632 (66.3)	<0.01	1.40 (1.05–1.88)	1.10 (0.78–1.57)
Fisher person	33 (8.9)	338 (91.1)		0.37 (0.24–0.57)	0.36 (0.22–0.60)
Other	336 (26.8)	919 (73.2)		1.12 (0.83–1.50)	1.09 (0.77–1.57)
Sex worker	2 (0.8)	240 (99.2)		0.03 (0.01–0.14)	0.04 (0.01–0.18)
Student	36 (24.0)	114 (76.0)		Ref	Ref

* *PRR, Prevalence Rate Ratio; CI, Confidence Interval. Adjusted for all variables shown as well as month of encounter*.

CBDs made referrals to long-term contraceptives for 398 (9%) of the encounters with women. Referral to long-term contraceptives was 19.5% among women <19 years-old and 14.2% among 20–24 year-old women, and decreased from 9.6% among women aged 25–29 years to 6.7% among women aged 40 years and older (*p* < 0.001; data not shown). Referral for long-term contraception was made for 12.3% of married women, compared to 7.4% of single women and 3.3% of widowed women (*p* < 0.001). Among women reporting no previous FP practices, 12.8% received referral to long-term contraception compared to 9.5% with prior FP use (*p* = 0.005). Overall, distribution of oral contraceptives or referral to long-term contraception was given to 34.5% of women, and this was 43.2% among women with no previous FP use.

### Factors Associated With Male Condom Distribution

Male condoms were distributed at 73% of client encounters, though this was at 94% of encounters with men and only 46% of the encounters with women (Table 2). The median number of individual condoms distributed at an encounter was 20. In multivariable modeling (Table 4), men were twice as likely to receive condoms than women (*p* < 0.001), with little variation in condom distribution by age. Single and widowed clients were more likely to have received condoms compared to married clients (*p* < 0.001). Sex workers and fishers were 86 and 18% more likely to receive condoms, respectively. There was interaction with gender and all covariates (*p* < 0.05). Women were less likely to receive condoms whereas men were more likely to receive them. Single and widowed women were more likely to receive condoms whereas single and widowed men were less likely to receive condoms. Among those having never used FP methods before, women were 13% less likely to receive condoms and men were 14% more likely (data not shown).

**Table 4:** Results of multivariable log-binomial regression: factors associated with receiving any male condoms.

	**Received male condoms**		**Chi-squared *P*-value**	**Crude PRR (95% CI)**	^*^Adjusted PRR (95% CI)
					***N* = 10,212**
	**Yes, *N* = 7,439 *n* (%)**	**No, *N* = 2,798 *n* (%)**			
**Gender**					
Male	5,385 (93.6)	368 (6.4)	<0.01	Ref	Ref
Female	2,054 (45.8)	2,430 (54.2)		0.49 (0.47–0.51)	0.48 (0.45–0.49)
**Age in years**					
13–19	495 (79.8)	125 (20.2)	<0.01	1.00 (0.95–1.05)	0.92 (0.87–0.98)
20–24	1,809 (72.3)	694 (27.7)		0.91 (0.87–0.94)	0.92 (0.88–0.97)
25–29	2,024 (71.2)	820 (28.8)		0.89 (0.86–0.94)	0.96 (0.92–1.00)
30–34	1,409 (70.7)	585 (29.3)		0.89 (0.85–0.92)	0.99 (0.94–1.03)
35–39	884 (70.7)	367 (29.3)		0.89 (0.84–0.93)	1.00 (0.95–1.05)
40+	818 (79.8)	207 (20.2)		Ref	Ref
**Marital status**					
Married	4,499 (66.8)	2,232 (33.2)	<0.01	Ref	Ref
Single	2,685 (85.6)	451 (14.4)		1.28 (1.25–1.31)	1.22 (1.19–1.26)
Widowed	255 (68.9)	115 (31.1)		1.03 (0.96–1.11)	1.28 (1.16–1.40)
**Session type**					
Individual	4,781 (73.8)	1,702 (26.2)	<0.01	Ref	N/A, not in final model
Household	789 (51.5)	742 (48.5)		0.70 (0.66–0.74)	
Group	1,868 (84.1)	353 (15.9)		1.14 (1.11–1.17)	
**Family planning usage**					
Ever	5,481 (73.6)	1,967 (26.4)	<0.01	Ref	Ref
Never	1,947 (70.1)	830 (29.9)		0.95 (0.93–0.98)	0.97 (0.94–0.99)
**Environmental population served**					
Environmental protection	1,604 (64.9)	868 (35.1)	<0.01	Ref	Ref
Natural resource management	2,468 (82.2)	536 (17.8)		1.27 (1.22–1.31)	1.20 (1.14–1.25)
Agricultural management	2,013 (67.7)	961 (32.3)		1.04 (1.00–1.08)	0.99 (0.95–1.04)
Fisher fork support	1,354 (75.8)	433 (24.2)		1.17 (1.12–1.21)	1.14 (1.09–1.20)
**Target populations**					
Farmer/environmental	4,678 (73.8)	1,660 (26.2)	<0.01	1.00 (0.94–1.08)	0.96 (0.90–1.03)
Fisher person	599 (82.9)	124 (17.1)		1.13 (1.05–1.22)	1.18 (1.09–1.27)
Other	1,703 (65.3)	905 (34.7)		0.89 (0.94–1.08)	0.94 (0.88–1.01)
Sex worker	225 (91.5)	21 (8.5)		1.25 (1.15–1.34)	1.86 (1.70–2.05)
Student	227 (73.5)	82 (26.5)		Ref	Ref

* *PRR, Prevalence Rate Ratio; CI, Confidence Interval. Adjusted for all variables shown as well as month of encounter*.

### Factors Associated With Integrated Messaging

Integrated FP and environmental messaging occurred at 89% of client encounters and was similar across organizations (85.6–91.0%). The agricultural management CBO disseminated environmental-only information at just 12 encounters (0.41%; Table 5). Due to the small cell size, modeling of this variable produced wide confidence intervals and large effect sizes. Receiving integrated messaging or reproductive-health-only messaging compared to environmental-only was more likely for women than men (aPRR = 1.85 and aPRR = 2.14, respectively; Table 6). Generally, as age increased, the likelihood of integrated messaging increased, and reproductive-health-only messaging decreased. Client encounters with single persons and widows were less likely to receive an integrated or reproductive-health-only message compared to encounters with married clients. Compared to those with previous FP use, clients who had never used FP were less likely to receive integrated or reproductive-health-only messages compared to environmental-only.

**Table 5:** Bivariate analysis of integrated messaging, *N* = 10,183.

	**Message**		
	**Integrated**	**Reproductive-health-only**	**Environmental-only**
	***N* = 9,071**	***N* = 710**	***N* = 402**
	***n* (%)**	***n* (%)**	***n* (%)**
**Gender**			
Male	5,093 (89.1)	351 (6.1)	275 (4.8)
Female	3,978 (89.1)	359 (8.0)	127 (2.8)
**Age in years**			
13–19	516 (84.1)	70 (11.4)	27 (4.4)
20–24	2,191 (88.2)	205 (8.3)	88 (3.5)
25–29	2,518 (88.9)	205 (7.2)	111 (3.9)
30–34	1,784 (90.0)	124 (6.3)	75 (3.8)
35–39	1,135 (91.1)	73 (5.9)	38 (3.1)
40+	927 (90.6)	33 (3.2)	63 (6.2)
**Marital status**			
Married	5,990 (89.5)	454 (6.8)	250 (3.7)
Single	2,751 (88.2)	244 (7.8)	124 (4.0)
Widowed	330 (89.2)	12 (3.2)	28 (7.6)
**Session type**			
Individual	5,678 (88.1)	509 (7.9)	259 (4.0)
Household	1,354 (88.8)	134 (8.8)	37 (2.4)
Group	2,037 (92.2)	67 (3.0)	106 (4.8)
**Family planning usage**			
Ever	6,738 (90.6)	442 (6.0)	254 (3.4)
Never	2,321 (84.8)	268 (9.8)	148 (5.4)
**Environmental population served**			
Environmental protection	2,179 (88.9)	182 (7.4)	89 (3.6)
Natural resource management	2,672 (89.4)	121 (4.1)	197 (6.6)
Agricultural management	2,693 (91.0)	254 (8.6)	12 (0.4)
Fisher fork support	1,527 (85.6)	153 (8.6)	104 (5.8)
**Target populations**			
Farmer/environmental	5,816 (91.9)	306 (4.8)	206 (3.3)
Fisher person	653 (90.6)	11 (1.5)	57 (7.9)
Other	2,127 (82.8)	315 (12.3)	126 (4.9)
Sex worker	215 (87.8)	27 (11.0)	3 (1.2)
Student	248 (80.5)	50 (16.2)	10 (3.3)

**Table 6:** Results of multivariable multinomial logistic regression^*^: factors associated with integrated messaging, *N* = 10,156.

**Variables**	**Integrated vs. environmental-only**	**Reproductive-health-only vs. environmental-only**
	**Adjusted PRR (95% CI)**	**Adjusted PRR (95% CI)**
**Gender**		
Male	Ref	Ref
Female	1.85 (1.46–2.34)	2.14 (1.61–2.84)
**Age in years**		
13–19	1.11 (0.64–1.94)	2.80 (1.36–5.77)
20–24	1.30 (0.90–1.87)	2.56 (1.50–4.38)
25–29	1.25 (0.90–1.75)	2.18 (1.32–3.61)
30–34	1.31 (0.91–1.89)	2.09 (1.24–3.54)
35–39	1.55 (1.01–2.38)	2.43 (1.34–4.39)
40+	Ref	Ref
**Marital status**		
Married	Ref	Ref
Single	0.89 (0.67–1.18)	0.74 (0.53–1.05)
Widowed	0.42 (0.27–0.66)	0.26 (0.12–0.55)
**Session type**		
Individual	Ref	Ref
Household	1.42 (0.97–2.08)	1.39 (0.90–2.14)
Group	1.21 (0.94–1.55)	0.46 (0.32–0.66)
**Family planning usage**		
Ever	Ref	Ref
Never	0.52 (0.41–0.66)	0.72 (0.54–0.97)
**Environmental population served**		
Environmental protection	Ref	Ref
Natural resource management	0.48 (0.34–0.68)	0.64 (0.41–0.99)
Agricultural management	9.05 (4.62–17.7)	16.5 (8.11–33.6)
Fisher fork support	0.58 (0.42–0.80)	1.27 (0.86–1.87)
**Target populations**		
Farmer/environmental	1.52 (0.73–3.17)	0.46 (0.21–1.02)
Fisher person	0.66 (0.30–1.44)	0.06 (0.02–0.17]
Other	0.73 (0.35–1.55)	0.69 (0.31–1.55)
Sex worker	2.00 (0.51–7.84)	2.55 (0.60–10.9)
Student	Ref	Ref

* *Observation-level analysis with cluster-based variance estimation at level of individual*.

### Client Exit Survey

Overall, 87 clients completed the exit interview, representing 12% of the 724 unique clients seen over the 2-month period the exit survey was offered. Participants were more likely to be female (58%) and similar in age (29 compared to 28 years) to the total program population. In general, respondents' reports confirmed program data regarding FP commodities received: 27% contraceptive pills among women, 27% of women with referral for long-term contraception, 75% receiving male condoms among all clients, 91% integrated messaging among all clients. Asked to rate the CBD they worked with, scores (*n* = 85) ranged from 5 to 10, with median “10” (64% reported “10”) and only 9 (11%) reported a score < “8”; all 85 respondents said they would return to this CBD again. Regarding the environmental programs, overall 84% of exit interview respondents reported receiving any environmental commodity or referral: 82% water and sanitation, 71% agricultural, 55% agroforestry, and 26% fishing.

## Discussion

Analysis of our PHE program demonstrates that most (89%) clients received integrated FP and environmental messages. Women were more likely than men to receive the integrated messages, but more than half (56%) of clients were men. This is a hallmark of the PHE approach, which has been demonstrated to be an effective method of reaching more men than would have been reached through traditional FP programs, in addition to improving FP uptake and practices (3, 12–14). This is critically important in Kenya and other sub-Saharan countries where men often play a primary decision-making role in the decision to adopt and sustain FP methods, and where men's knowledge is positively associated with female partner FP uptake (15–18). Our analyses identified actionable factors that could lead to improved program outcomes.

Analysis of the program also demonstrated FP integration with (1) male condoms distributed at 73% of client encounters and (2) contraceptive pills distributed at 28% encounters with women. Among women reporting no previous FP practices, 32% received contraceptive pills and this may represent new client coverage, though overall, only 36% of women received contraceptive pills (28%) or were referred for long-term methods (8%). Contraceptive distribution and FP messaging were more likely in individual sessions. This suggests that clients should be offered options for session type (individual, group, household) or set aside times for individual consultation during group sessions to reduce barriers for sensitive topics. Some target populations were less likely to receive contraception (e.g., fisher folk, sex workers). While some CBDs and program staff reported anecdotally that most sex workers were already receiving FP commodities, it is unclear why fisher folk women were less likely to receive contraceptive pills. There are numerous factors associated with uptake of contraceptives among women, and a broad literature review finds one key factor is the role of the male sex partner (19). Male partner support increases the uptake of contraceptives, and conversely, lack of partner approval can reduce the odds of contraceptive uptake (20–22), which we confirm in our study of couples in Kisumu, Kenya (23). Thus, in the current study, this may also contribute to explanation of lower uptake among women in household or group sessions, if their partners were not supportive.

Across the three CBOs and one NGO, there was substantial variability in the distribution of oral contraceptives (at 17–52% of encounters) and male condoms (at 65–82% of encounters), though integrated messaging was conducted consistently (at 86–91% of encounters). Though we did not collect this information as analyzable data, the main programmatic challenge reported by CBDs was erratic supplies of the FP commodities (“stockouts”). Stakeholder meetings between program staff and health facility staff identified recommendations to reduce stockouts: improved logistics and planning at the facility level, expanded budgeting at the county and national level, and strongly prioritizing consistent supply of FP commodities to reduce negative impacts on health. These recommendations are in keeping with studies demonstrating the impact of improved budgeting, planning, and logistics on reducing FP commodities stockouts (24, 25). Organizational or community characteristics may have impacted success in achieving program indicators. Previous PHE evaluations associate success with shared understanding and partnership with Ministry of Health workers, social marketing and support from local social institutions, inclusion of both male and female CBDs and younger PHE practitioners to appeal to adolescents and young adults (14, 26). In the future, measuring organizational level factors such as these and provision of interim assessment and feedback may mitigate sub-optimal distribution of commodities.

### Limitations, Strengths, and Recommendations

Our client encounter log lacked some measures that would enable more intensive evaluation of the program. USAID guidance for monitoring and evaluating PHE programs (27) recommends several individual and community level measures of population (e.g., percent of reproductive age women who were clients of a community-based distributor, outcomes of referrals), health (e.g., percent of households with improved water, sanitation and hygiene), environment (e.g., percent of farmers/fishers who adopt improved agricultural/marine practices), and integration indicators (e.g., percent of population within a target/project area receiving all three PHE elements) to evaluate effectiveness, though this requires long-term monitoring and resource investment in capturing these measures. In addition, the encounter logs only captured quantitative data and our results do not provide qualitative data to contextualize the findings. Future studies should include measures of quality and quantity of messaging, satisfaction with messaging and contraceptives, as well as additional characteristics of individuals, which could directly foster program improvement. Because of the large scale of the program, we recommend monitoring a small proportion of randomly selected clients to validate program indicators at the individual level. A strength of our program is that we measured integrated messaging, capture of non-traditional audiences, and the number of PHE educational sessions provided in the target community. Our exit survey confirmed high levels of referral and commodities distribution for environmental programming. In keeping with the goals of PHE, we created new partnerships that linked organizations from different sectors. Given that PHE programs already have demonstrated effectiveness, we did not make use of a control area; rather, our goal was to implement a PHE program on a community scale. To observe the impact on fertility practices of this PHE program and others like it, we recommend sustained programming with population level surveillance, preferably capitalizing on existing Demographic Health Surveys.

## Conclusions

The SHAPE-LVB program demonstrated community-level scale-up integrating FP commodities distribution and messaging into routine environmental conservation. Program outcomes may be strengthened by tailoring the session types, increased mobilization of integrated services among men, widows, and those without prior FP, and assessment of organization-level barriers and facilitators.

## Data Availability Statement

The original contributions presented in the study are included in the article/Supplementary Material, further inquiries can be directed to the corresponding author/s.

## Ethics Statement

The analysis of SHAPE-LVB program data was determined exempt (did not meet the definition of human subject research) by the University of Illinois at Chicago School of Public Health.

## Author Contributions

EO, MO, HW, and DO were involved in program design. EO provided program oversight and drafted the manuscript. EO, KS, DO, and SM contributed to the design of exit survey measures. SM designed and advised on the statistical analysis. KS conducted statistical analyses and table preparation and presentation of data, and wrote statistical analysis section of manuscript. MO and HW provided quality control and developed and conducted trainings. GO led design and implementation of all data capture and performed data cleaning and coordination. All authors contributed to the article and approved the submitted version.

## Conflict of Interest

The authors declare that the research was conducted in the absence of any commercial or financial relationships that could be construed as a potential conflict of interest.
